# Mr. Luscombe, on the Influenza

**Published:** 1803-08-01

**Authors:** Edward T. Luscombe

**Affiliations:** Bexhill Barracks


### To Dr. BRADLEY.

My dear Sir,

MEDICAL practitioners of the first eminence appearing divided in their opinion on the question now so frequently agitated, Whether the epidemic, which lately prevailed, was *contagious or not?* I have (agreeably to the invitation given your Correspondents to communicate the result of their experience in this disease), transmitted an account of its appearance and progress in the cavalry barrack at East Bourn, occupied by a troop of the second regiment of dragoons. If you think the facts it contains will assist your readers in forming an opinion on this interesting question, you will have the goodness to insert it in the next number of your useful publication.

I am, &c.

*Bexhill Barracks, June* 16, 1803.

EDWARD T. LUSCOMBE.

---

The cavalry barracks at East Bourn, occupying a square of about four acres, is situated within a mile of the sea, and one hundred and fifty yards of the town; but being built on an acclivity, is more exposed than East Bourn; particularly to the rage of the south-west winds, which are very prevalent, and blow at times, over Beachy Head, with tremendous violence.

The officers' apartments are separated by a thick brick wall from those appropriated to the accommodation of the non-commissioned officers and privates; the latter, consisting of ten rooms, are on the same floor.

During the month of March last, the influenza was very prevalent at East Bourn, and was epidemic in the town and neighbourhood some time before it made its appearance in the barracks. On

On March 19th, in the evening, two private dragoons, in rooms No. 4 and 7, complained of violent pain in the head over the eyes, and every symptom of influenza, as described by Dr. R. Pearson, who all followed the treatment laid down in his publication: one was discharged to his duty on March 24, the other not until April 2; cough and debility resisting the usual remedies.

A third dragoon, in room No. 3, was attacked on March 21st, and remained incapable of doing his duty until the 25th; a fourth private was also attacked on that day in room No. 6, and remained on the sick list until the 27th. The farrier's wife, in room No. 4, and a child (who was, if I recollect rightly, in the same room) also went through the disease about the same time. All those persons continued in the barrack rooms; an hospital not having been then built.

The subjoined account will shew at one view, the number of persons in each room, and the proportion those attacked bore to those who escaped the disease.

I am not prepared to give it as my decided opinion, that the epidemic which lately prevailed, *was not contagious*; but from its appearance in East Bourn barracks, and all the information that I have been able to collect, I am inclined to think that it was not. And I believe that *its cause* must be sought in the state and variations of the atmosphere, and great and sudden changes of temperature.

*An Account of the Number of Persons who were attacked by or escaped the Influenza in the non-commissioned Officers and private Soldiers' Rooms, East Bourn Barracks, March,* 1803.

| | Men in each room. | Women in each room. | Children in each room. | Total of persons in each room. | Men attacked by the disease. | Women attacked. | Children attacked. | Total of persons attacked by the disease. | Total of persons who escaped the disease. |
|---|---|---|---|---|---|---|---|---|---|
| Quarter-master's room | 1 | 1 | 2 | 4 | — | — | — | — | 4 |
| Barrack serjeant's do. | 1 | — | — | 1 | — | — | — | — | 1 |
| Serjeants room | 3 | 1 | 2 | 6 | — | — | — | — | 6 |
| Soldiers room, No. 1. | 7 | 1 | 1 | 9 | — | — | — | — | 9 |
| ———— No. 2. | 7 | 1 | 1 | 9 | — | — | — | — | 9 |
| ———— No. 3. | 4 | 1 | 1 | 6 | 1 | — | — | 1 | 5 |
| ———— No. 4. | 8 | 1 | 1 | 10 | 1 | 1 | 1 | 3 | 7 |
| ———— No. 5. | 8 | — | — | 8 | — | — | — | — | 8 |
| ———— No. 6. | 8 | 1 | 1 | 10 | 1 | — | — | 1 | 9 |
| ———— No. 7. | 8 | 1 | 1 | 10 | 1 | — | — | 1 | 9 |
| Total | 55 | 8 | 10 | 73 | 4 | 1 | 1 | 6 | 67 |

